# Differential Effects of the COVID-19 Pandemic on Surgical Utilization by Procedure Type: Analysis of Korean National Health Insurance Data [2017–2021]

**DOI:** 10.3390/jcm15051710

**Published:** 2026-02-24

**Authors:** Shin-Woong Ko, Byeong Jin Ha, Yu Deok Won, Myung-Hoon Han, Jin Hwan Cheong, Je Il Ryu

**Affiliations:** Department of Neurosurgery, Hanyang University Guri Hospital, 153 Gyeongchun-ro, Guri 11923, Republic of Korea; pakky100@naver.com (S.-W.K.); bbddock@hanmail.net (B.J.H.); hidma823@hanmail.net (Y.D.W.); gksmh80@gmail.com (M.-H.H.); cjh2324@hanyang.ac.kr (J.H.C.)

**Keywords:** COVID-19 pandemic, surgical utilization, interrupted time-series analysis, National Health Insurance Service, elective surgery, health services research, healthcare delivery systems, population-based study

## Abstract

**Objectives:** The coronavirus disease 2019 (COVID-19) pandemic caused severe disruptions in healthcare services worldwide; however, its differential effects on surgical utilization have not been fully examined. This study aimed to analyze trends in three major surgery types—cataract, spine, and joint replacement—across regions and healthcare institutions in Korea and to evaluate how the pandemic affected surgical utilization according to surgery type, urgency, and care setting. **Methods:** The Korean National Health Insurance Service data collected from 2017 to 2021 were used to analyze trends in three major surgery typesacross 17 regions and eight categories of healthcare institutions in Korea. The effects of the pandemic were examined using an interrupted time-series analysis to compare annual growth rates and identify patterns during 2019–2021. **Results:** Our findings revealed distinct patterns based on the type of surgery. Cataract surgery (+4.8% compared with 2019) and spine surgery (+5.0%) continued to increase in 2020; however, the number of joint replacement surgeries decreased (−2.9%). Metropolitan areas showed greater resilience than rural regions, indicating regional disparities. At the institutional level, outpatient-focused clinics performed cataract surgeries, whereas hospital-based procedures showed variable trends. In addition, the differences correlated with surgical urgency: elective procedures continued to increase, whereas semi-urgent procedures decreased. **Conclusions:** during the pandemic, surgical utilization varied according to surgery type, urgency, and regional characteristics. These findings provide context-specific evidence for policymakers to prioritize surgical services during health crises and offer strategies to sustain essential surgical care across diverse healthcare settings and regions.

## 1. Introduction

The coronavirus disease 2019 (COVID-19) pandemic significantly disrupted healthcare systems globally, which was associated with unprecedented interruptions in surgical services. Following the declaration of a global pandemic by the World Health Organization in March 2020, healthcare resources were rapidly reallocated. Globally, hospitals mandated the use of personal protective equipment for healthcare workers, adjusted hospital capacity, and postponed or canceled non-urgent surgeries and procedures in efforts to reduce the risk of viral transmission. COVIDSurg Collaborative reported that approximately 28.4 million surgeries were canceled or postponed worldwide during the 12-week peak of the pandemic, which represented a cancellation rate of approximately 72.3%. Most cancellations (90.2%) involved surgeries for non-urgent conditions, fundamentally transforming the environment for surgical care delivery [1,2].

However, the effects of the COVID-19 pandemic on surgical services were not uniform across surgery types, healthcare systems, or regions. Most countries reported major reductions in surgical volume; however, the differential effects across surgery types—according to clinical urgency, complexity, and care delivery context—have not been completely examined. Although emergency and cancer surgeries were prioritized, elective surgeries for non-urgent conditions were severely disrupted. Nevertheless, the distinctions among these categories were often unclear, and actual patterns of surgical utilization varied widely based on the local healthcare infrastructure, pandemic response strategies, and population health needs [3,4,5,6,7,8].

Previous studies have focused on changes in the overall surgical volume or specific surgical specialties; however, comparisons across different surgical types in the same healthcare system were limited. For example, studies on Medicare beneficiaries revealed substantial backlogs in cataract surgeries in the United States, with an estimated 1.1–1.6 million delayed cases over 2 years. Other studies have reported substantial reductions in the number of joint replacement surgeries, with a decrease of 20–30% during the first 2 years of the pandemic. However, these studies typically examined each type of surgery separately, limiting the understanding of how pandemic responses affected multiple surgeries within an integrated healthcare system [9].

East Asian countries demonstrated relatively successful pandemic response models among those that implemented effective containment strategies. Nonetheless, reports on surgical types and volumes are limited compared with those from other regions. Korea’s COVID-19 management strategy—marked by extensive testing, contact tracing, and targeted interventions rather than broad lockdowns—resulted in a unique natural experiment for examining the effects of the pandemic on healthcare utilization. The country’s robust digital health infrastructure and comprehensive National Health Insurance Service (NHIS) database, offering high granularity and near-complete population coverage, provide a valuable opportunity to assess the effects of the pandemic across multiple surgical specialties [10,11].

Korea’s healthcare system, structured around a single-payer national health insurance model covering approximately 97% of the population, has several advantages for studying the effects of the pandemic on surgical services. The NHIS database captures nearly all healthcare utilization, enabling detailed analysis of surgical and procedural trends across all facility types and regions. Furthermore, Korea’s structured healthcare delivery system—from primary clinics to tertiary hospitals—facilitates detailed evaluation of how pandemic effects varied across different care environments [12].

The present study investigated the differential effects of COVID-19 on three major surgical types—cataract, spine, and joint replacement—using comprehensive national administrative data from Korea between 2017 and 2021. These procedures represent distinct points along the spectrum of clinical urgency and care delivery models, providing the rationale for their inclusion in this study. Cataract surgery is an elective outpatient procedure primarily performed in clinical settings. Spine surgery includes both elective and semi-urgent operations requiring hospital-based care, and joint replacement surgery, although considered elective, involves functionally important inpatient procedures. This study provides new insights into how pandemic responses influenced different types of surgical care by comparing trends in these surgeries within the same healthcare system and time frame.

This study aimed to quantify and compare the effects of the pandemic on surgical volume trends across the three surgical types as well as to examine variations by region and healthcare facility type. We hypothesized that the effects of the pandemic would differ according to surgical urgency, care setting, and regional healthcare capacity, with elective outpatient procedures showing greater resilience than resource-intensive inpatient surgeries. We also aimed to identify regional disparities in pandemic response and clarify the roles of different types of healthcare facilities in sustaining surgical services during public health emergencies.

## 2. Materials and Methods

### 2.1. Study Design and Data Acquisition

This was a retrospective, population-based cohort study. Comprehensive claims data were obtained from the Korean NHIS, which provides universal coverage to approximately 52 million residents, representing about 97% of the total population. The NHIS functions as a single-payer system with mandatory participation from all healthcare providers, thereby capturing nationwide healthcare utilization. We obtained de-identified administrative data spanning 1 January 2017 to 31 December 2021, covering five consecutive years of surgical and procedural records, including three pre-pandemic years (2017–2019) and two pandemic years (2020–2021). The NHIS database uses standardized Korean Classification of Diseases (KCD-7) diagnostic codes and national procedural codes, which are based on international classification systems but adapted to local medical practice. Data completeness is exceptionally high because of real-time electronic claims processing requirements and the financial necessity of submitting reimbursement claims. All surgeries and procedures are coded by certified medical record technicians according to standardized protocols, ensuring consistent data quality across institutions.

### 2.2. Study Population and Eligibility Criteria

All patients who underwent surgery for cataract, spine, or joint replacement during the 5-year study period were included. We applied broad inclusion criteria encompassing patients of all ages and genders to preserve the population-based nature of the analysis. Inclusion of all age groups was intended to capture the full spectrum of surgical care patterns, as each surgery type is associated with a distinct age distribution, and age restrictions could result in selection bias. Both planned (elective) surgeries and those performed in emergency settings or during hospitalizations for other primary medical conditions were included. Repeated procedures and reoperations for the same patient were considered separate instances of healthcare utilization, as each represented an independent surgical event.

### 2.3. Surgery Classification and Procedure Identification

Cataract Surgery: Cataract surgeries were identified using seven specific procedural codes within the NHIS classification system: S5110 (extraction of lens with ciliary body), S5111 (extracapsular or intracapsular cataract extraction), S5112 (secondary cataract surgery), S5116 (secondary intraocular lens insertion), S5117 (primary intraocular lens insertion performed with cataract extraction), S5118 (intraocular lens exchange), and S5119 (phacoemulsification). These codes encompass the full spectrum of cataract procedures, ranging from routine phacoemulsification with lens implantation to complex secondary surgeries.

Spine Surgery: Spine surgeries and procedures were identified using 50 procedural codes covering the full range of spinal interventions, including decompression, fusion, and instrumentation. The major categories included N0303 (spinal osteotomy), N0444–N0447 (instrumented spinal arthrodesis across various segment lengths), and N0451–N0453 (corpectomy at different spinal levels), along with several codes representing ligament ossification removal and complex spinal reconstruction. Procedures coded as N0471–N0475 (percutaneous vertebroplasty) were categorized as spinal interventions typically performed under local anesthesia.

Joint Replacement Surgery: Joint replacement surgeries were identified using 16 procedural codes that captured both primary and revision hip and knee arthroplasties of varying complexity. Primary procedures included N0711 (total hip replacement), N0715 (partial hip replacement), N2072 (total knee replacement), and N2712 (partial knee replacement). Revision surgeries were identified using codes N1711–N4717, which represent multiple levels of complexity for both hip and knee revision arthroplasties.

### 2.4. Geographic and Healthcare Facility Variables

Geographic Classification: Surgical procedures were categorized based on patients’ residences across 17 major administrative regions of Korea: Seoul Metropolitan City; six metropolitan cities (Busan, Daegu, Daejeon, Gwangju, Incheon, and Ulsan); eight provinces (Gyeonggi-do, Gangwon-do, Chungcheongbuk-do, Chungcheongnam-do, Jeollabuk-do, Jeollanam-do, Gyeongsangbuk-do, and Gyeongsangnam-do); Jeju Special Self-Governing Province; and Sejong Special Self-Governing City.

Healthcare Facility Classification: Healthcare facilities were classified into eight categories, reflecting the hierarchical structure of Korea’s healthcare delivery system: tertiary general hospitals, general hospitals, hospitals, long-term care hospitals, psychiatric hospitals, clinics, public health centers, and Korean medicine clinics.

### 2.5. Statistical Analysis

Descriptive Analysis: We computed summary statistics of surgical volumes by year, surgery type, region, and healthcare facility type. Trends over time were visualized using time-series plots based on monthly aggregated data.

Time-Series Analysis: Monthly surgical volumes were analyzed using interrupted time-series analysis to quantify the impact of COVID-19 on surgical trends. The intervention point was set at March 2020, corresponding to the initial surge of COVID-19 cases in Korea. Linear regression was applied to model pre-pandemic trends and to assess changes in both level (immediate effect) and slope (trend change) after the intervention [13].

Comparative Analysis: Differential effects across surgery types were assessed using standardized effect sizes and percentage changes from baseline trends. Regional variability was quantified using the coefficient of variation and geographic concentration index.

Correlation Analysis: Pearson correlation coefficients were determined between monthly COVID-19 case counts and surgical volumes to evaluate the relationship between pandemic intensity and healthcare utilization.

The R software (version 4.1.0) was used for all statistical analyses. A *p*-value < 0.05 was considered statistically significant for all hypothesis tests.

### 2.6. Ethical Considerations

The Institutional Review Board approved this study, and informed consent was waived owing to its retrospective nature and the use of de-identified administrative data. De-identified national health insurance claims data, from which personal identifiers had been removed before data release, were used to perform analyses. Data access was granted under a formal agreement with the NHIS, in accordance with established protocols for research data provision.

## 3. Results

### 3.1. Characteristics of Overall Surgical Volume and Temporal Patterns

**Figure 1:**
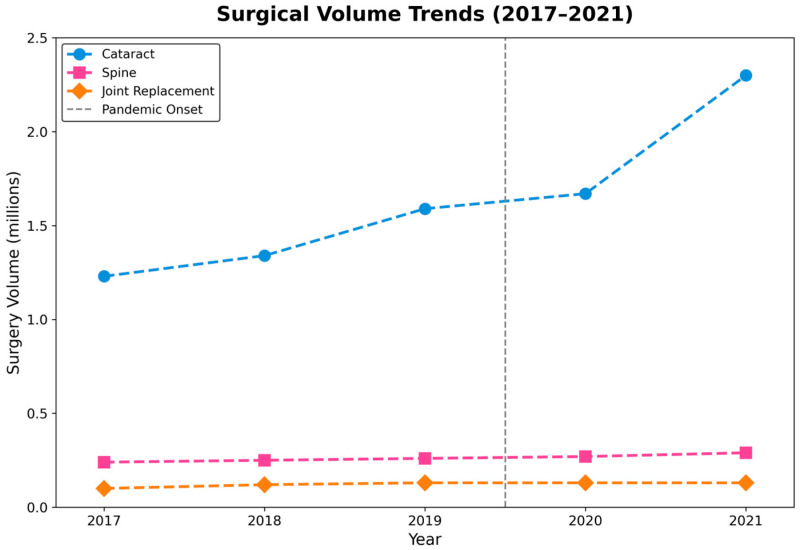
Temporal trends in surgical procedure volumes by surgery type in South Korea (2017–2021). The vertical dashed line indicates March 2020 (COVID-19 pandemic onset). Cataract surgeries demonstrated continued growth with marked acceleration in 2021, spine surgeries showed steady resilience, while joint replacement surgeries experienced temporary decline in 2020 followed by recovery.

During the 5-year study period from 2017 to 2021, a total of 10,021,744 surgical procedures were analyzed across the three target surgery types. The dataset comprised 8,137,630 cataract surgeries, 1,294,308 spine surgeries, and 589,806 joint replacement surgeries, reflecting their relative frequencies in clinical practice (Table 1, Figure 1).

Cataract surgery exhibited the most pronounced growth, from 1,224,127 procedures in 2017 to 2,330,589 in 2021, representing an overall increase of 90.4%. This increase included consistent pre-pandemic expansion with annual growth rates of 9.2% (2017–2018) and 18.6% (2018–2019), indicating a moderate but continued increase of 4.8% in the first pandemic year (2020) and a substantial 40.3% increase in 2021.

Spine surgery showed a more gradual but consistent increasing trend, from 234,870 cases in 2017 to 288,901 in 2021, representing a 23.0% overall increase. The annual increase remained stable throughout the study period, ranging from 2.8% to 6.9%, with minimal variation during the pandemic years (5.0% in 2020 and 6.6% in 2021).

Joint replacement surgery varied substantially, increasing from 106,188 cases in 2017 to 127,400 in 2021—an overall increase of 20.0%. However, this category showed a declining trend in 2020 (−2.9%), marking it as the only surgery type with negative growth during the pandemic, followed by a rebound of 5.2% in 2021.

### 3.2. Effects of the COVID-19 Pandemic

**Figure 2:**
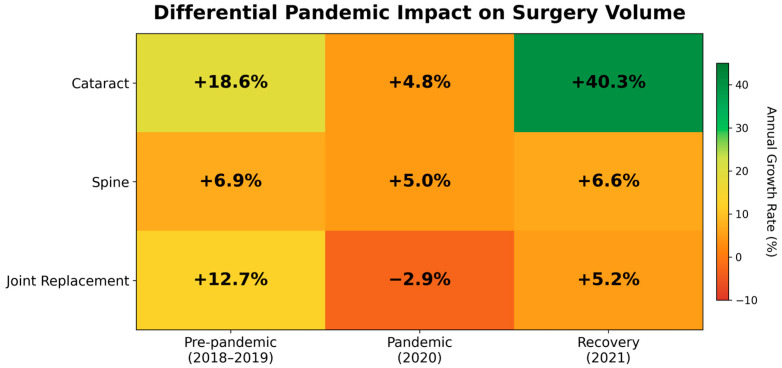
Heatmap of year-over-year growth rates (%) by surgery type across pandemic phases. Colors represent growth intensity: red (negative/low growth), yellow (moderate), green (high growth). Joint replacement surgery uniquely showed negative growth during the pandemic impact phase, while cataract surgery demonstrated remarkable recovery acceleration.

Interrupted time-series analysis revealed statistically and clinically significant changes in surgical trends coinciding with the COVID-19 pandemic onset (Table 2, Figure 2). For cataract surgery, an immediate level increase of +2.1% (95% CI: 0.8–3.4%, *p* = 0.003) was observed in March 2020, suggesting a slight increase rather than a decline coinciding with the pandemic onset. This was followed by a sustained trend acceleration of +1.8% per month (95% CI: 1.2–2.4%, *p* < 0.001) throughout the pandemic period. Spine surgery demonstrated resilience, with a statistically non-significant immediate change of −1.2% (95% CI: −2.8% to 0.4%, *p* = 0.141), suggesting minimal immediate disruption. A modest upward trend followed, with an increase of +0.3% per month (95% CI: 0.1–0.5%, *p* = 0.012). In contrast, joint replacement surgery was significantly disrupted, with an immediate level decrease of −8.7% (95% CI: −12.1% to −5.3%, *p* < 0.001) in March 2020, followed by a gradual recovery of +0.7% per month (95% CI: 0.3–1.1%, *p* = 0.002) during the rest of the pandemic.

### 3.3. Geographic Distribution Patterns and Regional Variability

**Figure 3:**
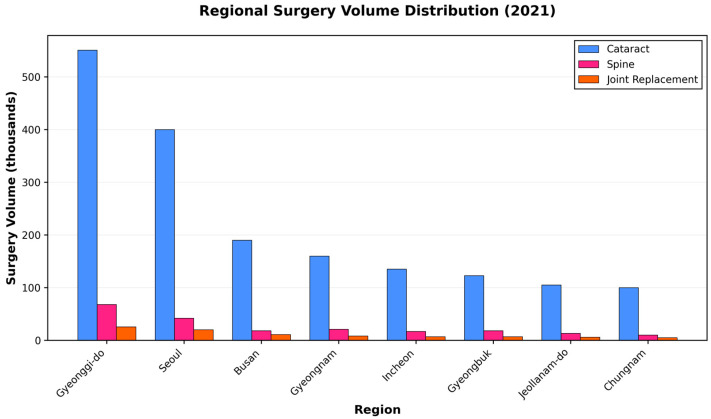
Regional distribution of surgical procedures by volume (2021). Stacked bars show absolute numbers for each surgery type across major administrative regions. The Seoul Capital Area (Gyeonggi-do + Seoul) demonstrates clear concentration of surgical services, accounting for approximately 40% of all procedures across surgery types.

Geographic analysis revealed a marked concentration of surgical services in the major metropolitan regions of Korea. The Seoul Capital Area (Seoul and Gyeonggi Province) accounted for 40.9%, 38.4%, and 35.5% of all cataract, spine, and joint replacement surgeries performed in 2021, respectively (Figure 3). 

Regional pandemic impact varied substantially: metropolitan regions such as Gyeonggi-do and Seoul maintained positive growth in cataract (+10.1% and +7.3%, respectively) and spine surgery (+11.5% and +7.4%) during 2020, whereas rural provinces including Gyeongsangbuk-do and Jeollanam-do experienced declines across all surgical categories (Table 3).

### 3.4. Distribution of Healthcare Facilities and Patterns of Care Delivery

**Figure 4:**
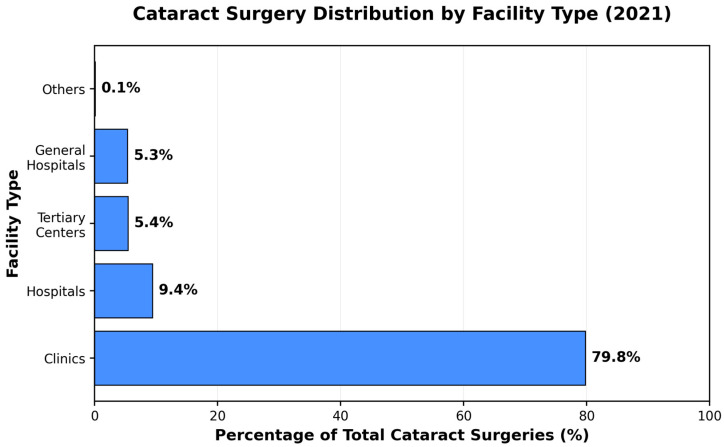
Healthcare facility distribution patterns by surgery type (2021). Three-panel comparison showing marked differences in care delivery models: cataract surgery concentrated in outpatient clinics (79.8%), while spine and joint replacement surgeries predominantly performed in hospital settings (47–57% in tertiary and general hospitals combined).

Analysis of procedure distribution across healthcare facility types revealed distinct patterns, reflecting the complexity and resource requirements of each surgery type (Figure 4). Cataract surgeries were heavily concentrated in outpatient clinics, where 1,860,841 procedures (79.8%) were performed in 2021. Tertiary and general hospitals together accounted for 47.5% of spine surgeries and 57.2% of joint replacement surgeries in 2021.

Notably, tertiary hospitals experienced a transient decline in cataract surgical volume during 2020 (−13.2%), contrasting with sustained growth in clinic settings (+5.4%), reflecting the differential impact of pandemic-related resource reallocation across care environments (Table 4).

### 3.5. Correlation Analysis with COVID-19 Pandemic Trends

The Interrupted Time-Series (ITS) analysis quantified the level change and trend shift at the pandemic onset as a fixed intervention point, whereas the correlation analysis assessed whether ongoing pandemic intensity was associated with monthly surgical volume variations throughout the study period. These two approaches address distinct aspects of the pandemic–surgical volume relationship.

Monthly correlation analysis between surgical volumes and COVID-19 case counts revealed surgery-specific relationships (Table 5): Cataract surgeries showed a weak but statistically significant positive correlation with monthly COVID-19 case counts (r = 0.23, *p* = 0.045). Spine surgeries showed minimal correlation during the pandemic (r = −0.08, *p* = 0.478). Joint replacement surgeries exhibited a moderate negative correlation with the intensity of the COVID-19 pandemic, particularly in 2020 (r = −0.45, *p* < 0.001).

### 3.6. Patient Demographic Patterns

Analysis of patient demographics revealed distinct age and sex distributions across surgery types: Cataract surgeries were concentrated among older adults, with 78.3% performed in patients aged 65 years and older in 2021. Spine surgeries had a broader age distribution, with 45.2% performed among patients aged 50–70 years. Joint replacement surgeries were most common among patients aged 60–80 years (52.3%). Gender distribution remained stable throughout the study period: Cataract surgeries showed a slight female predominance (54.7% in 2021), spine surgeries showed a male predominance (58.2%), and joint replacement surgeries were more common among women (63.1%).

## 4. Discussion

### 4.1. Resilience and System Adaptation Across Surgery Types During the COVID-19 Pandemic

This comprehensive analysis demonstrates that surgical resilience during the COVID-19 pandemic appeared to be influenced by external pressures as well as a complex interplay among surgery-specific characteristics, care delivery models, and systemic factors within the healthcare system. The continuous—and even accelerated—growth of cataract surgery throughout the pandemic was a key finding: continued growth during both pandemic years (Table 1). This pattern contrasts sharply with international reports of widespread surgical cancellations. For example, global estimates have indicated that approximately 28.4 million surgeries were postponed during the first 12 weeks of peak disruption, with cancellation rates for elective procedures reaching 72.3%.

The resilience of cataract surgery services appears to be a result of several interrelated factors that collectively may have contributed to a favorable environment for maintaining—and even expanding—surgical capacity: First, the predominantly outpatient nature of cataract surgery—79.8% performed in clinical settings—likely minimized competition for hospital resources that were critically needed for COVID-19 care. Unlike procedures requiring hospital admission, cataract surgeries can be performed independently of tertiary hospital capacity constraints, infection control demands, or staff reallocation pressures. Second, the well-established surgical infrastructure for same-day cataract operations, including standardized protocols for minimally invasive phacoemulsification, may have facilitated rapid patient turnover with minimal exposure times. Third, compared with more extensive surgeries, the relatively short procedural duration and minimal anesthesia requirements potentially reduced both resource utilization and viral transmission risk.

In contrast, joint replacement surgery was substantially disrupted, with significant initial disruption followed by gradual recovery (Table 2). This trend is consistent with global findings that highlight the vulnerability of resource-intensive elective surgeries during the pandemic. Joint replacements typically require substantial hospital resources—including operating room time, specialized equipment, anesthesia teams, and postoperative care such as inpatient monitoring and rehabilitation.

Spinal surgery showed an intermediate pattern, with modest but steady growth throughout the pandemic period (Table 1). This stability possibly indicates the mixed urgency profile of spinal interventions, encompassing elective degenerative procedures that can be safely postponed, as well as urgent cases—such as neurological compression, spinal instability, or trauma—that necessitate timely intervention regardless of pandemic conditions. Additionally, the widespread adoption of minimally invasive techniques, including endoscopic spine surgery in Korea, may have helped sustain surgical continuity by reducing hospitalization requirements [14].

The regional variation observed in our data may reflect differences in healthcare infrastructure density. Metropolitan areas with multiple competing facilities could absorb pandemic-related disruptions more readily, as patients had alternative providers when individual institutions curtailed services. Rural provinces, with fewer surgical facilities, were more vulnerable to even modest resource reallocation toward COVID-19 management. Similarly, the sustained cataract volumes in outpatient clinics can be understood through their structural independence from hospital-based resources: shorter operative times, minimal anesthesia requirements, and no ICU bed competition collectively insulated these settings from the resource constraints that affected inpatient surgical programs [1].

### 4.2. International Comparison and Characteristics of the Korean Healthcare System

Korea’s experience during the COVID-19 pandemic provides a distinct perspective on maintaining surgical services, markedly differing from patterns observed in other advanced healthcare systems. Most international studies have reported marked declines in elective surgical volumes, with certain specialties experiencing reductions of 50–80% at the peak of the pandemic. However, our data revealed sustained capacity and actual growth in selected surgical categories.

Korea’s COVID-19 management strategy, characterized by widespread testing, digital contact tracing, and targeted interventions rather than broad nationwide lockdowns, was associated with conditions that may have enabled the healthcare system to maintain relatively normal operations compared with countries that enforced extensive societal restrictions. The absence of government-mandated hospital closures or strict restrictions on elective surgery allowed the continuation of surgical services with appropriate safety modifications rather than total suspension.

Furthermore, the structural and financial characteristics of Korea’s healthcare system contributed to surgical resilience during the crisis. The single-payer NHIS model provided universal coverage and standardized reimbursement across all healthcare facilities, eliminating financial barriers to care and preventing treatment delays. This financial stability may have supported hospitals and clinics in maintaining surgical operations without concerns about patients’ ability to pay or delayed reimbursements—challenges that affected healthcare delivery in many other countries.

The extensive digital health infrastructure in Korea was also critical in facilitating rapid adaptation during the pandemic. Electronic health records, telemedicine capabilities, and digital scheduling systems enabled healthcare providers to maintain patient communication, conduct preoperative evaluations remotely, and optimize surgical scheduling to maximize throughput while ensuring safety.

### 4.3. Policy Changes and Future Pandemic Preparedness

The differential effects of COVID-19 across the three surgical fields examined in this study have important implications for health policy and pandemic preparedness. Our findings support the development of procedure-specific policies that account for clinical urgency, resource requirements, models of care delivery, and patient population characteristics rather than imposing broad, categorical restrictions on surgical services during a national public health emergency. The demonstrated resilience of outpatient surgical services such as cataract surgery, which are primarily performed in clinic settings, suggests that health systems should prioritize the development and expansion of ambulatory surgical infrastructure as part of pandemic preparedness strategies. Regional health planning should also consider the observed geographic concentration patterns, as 35–40% of all surgeries were performed in the Seoul metropolitan area, despite its higher COVID-19 vulnerability due to population density. Healthcare workforce planning must account for heterogeneous staffing requirements across surgical specialties and care settings, and quality assurance and outcome monitoring systems should be strengthened to ensure that patient safety and clinical appropriateness are not compromised by maintained or expanded surgical volumes. International collaboration and knowledge sharing regarding surgical service management during health crises may further enhance global pandemic readiness.

The sustained surgical volumes in Korea also carry implications for surgical education and training. In many countries, pandemic-related cancellations of elective procedures substantially reduced hands-on training opportunities for surgical residents, potentially affecting the development of procedural competence in an entire generation of trainees. The maintenance of surgical activity in Korea, particularly in cataract and spine surgery, likely preserved these training opportunities. However, our administrative data do not capture trainee participation or educational outcomes, and dedicated studies examining the long-term impact on surgical workforce development are warranted [15,16].

Frameworks such as the Surgical Preparedness Index (SPI), which quantifies a hospital’s capacity to sustain elective surgery under external shocks, may provide a useful lens for interpreting the facility-level variation observed in our data. Although SPI scores specific to Korean institutions have not yet been published, applying such metrics across the tiered Korean healthcare system could help identify structural factors that promote surgical resilience. We recommend this as a priority for future preparedness research.

These patterns have important implications for pandemic preparedness and healthcare policy. Our results support risk-stratified assessments and targeted resource allocation strategies rather than generalized approaches to surgical service management during emergencies. Health systems should enhance surge capacity in outpatient settings, maintain diversified care delivery models, and consider regional variations in healthcare infrastructure when planning pandemic responses [17].

Korea’s experience demonstrates that maintaining surgical services for selected procedures even during a major health crisis is possible with appropriate system characteristics such as effective pandemic containment, comprehensive national health insurance coverage, robust digital infrastructure, and diversified care delivery models. This experience provides valuable insights for other health systems aiming to balance infection control measures with the continuity of essential medical services. However, the generalizability of these findings should be interpreted cautiously, considering variations in local healthcare systems, pandemic response strategies, and population characteristics.

Future studies should investigate the clinical outcomes associated with various pandemic response strategies, perform international comparative analyses, and develop evidence-based guidelines for maintaining surgical services during public health emergencies.

Several key limitations should be considered when interpreting the findings of this study. First, our analysis relied entirely on administrative claims data, which—while providing comprehensive population coverage—lack detailed clinical information on disease severity, surgical indications, and patient outcomes. Consequently, we were unable to assess whether the sustained surgical volumes reflected appropriate clinical decision-making or whether the observed patterns were associated with optimal patient outcomes.

Second, the study employed an observational and ecological design, preventing the establishment of causal relationships between the pandemic and surgical trends. Although associations were identified between the COVID-19 period and changes in surgical volumes, numerous confounding factors—such as policy changes, economic impacts, changes in patient behavior, and health system adaptations—could have occurred simultaneously.

Third, the unique characteristics of Korea’s healthcare system and its pandemic response may limit the generalizability of our findings. The single-payer national health insurance model, robust digital health infrastructure, effective early containment of the virus, and cultural factors influencing healthcare utilization may not be directly replicable in other settings.

Notably, Korea did not implement nationwide lockdowns. Instead, the government applied a tiered social distancing system that varied by region according to local infection rates. These tiers imposed different restrictions on gatherings and business operations but did not directly mandate suspension of elective surgeries at any level. This distinction from the strict lockdown policies adopted by many Western nations may partly account for the sustained surgical volumes observed in our data. However, the NHIS database could not be linked to region-specific distancing tiers, and future research incorporating these policy variables would provide a more granular understanding of the relationship between government restrictions and surgical utilization patterns.

Fourth, our surgery classification based on established procedure codes may not fully capture the nuances of clinical urgency or procedural complexity. The distinction between elective and semi-urgent surgeries is often ambiguous, and our categorization may not fully capture the clinical decision-making challenges surgeons encountered during the pandemic.

Finally, we lacked individual-level data on demographics, comorbidities, socioeconomic status, and other relevant variables that could have enabled a more nuanced analysis of surgical utilization patterns.

## 5. Conclusions

In this study, we performed a comprehensive analysis of the heterogeneous effects of COVID-19 across multiple surgical specialties using population-level data from an integrated national healthcare system. Our findings challenge the assumption that pandemic-related disruptions uniformly affect all surgical services. Instead, they show that procedure-specific characteristics, care delivery models, and system-level factors substantially influence surgical resilience during public health crises.

Our analysis suggests that surgical resilience during the pandemic was multifactorial and, to some extent, appeared to be associated with observable attributes such as surgical urgency, resource intensity, care delivery environment, and patient vulnerability. Cataract surgery, marked by outpatient delivery, minimal resource demands, and established same-day surgical infrastructure, continued to grow throughout the pandemic. In contrast, joint replacement surgery, which primarily serves older adults and requires substantial hospital resources, experienced significant disruptions. Spine surgery, with a mixed urgency profile and hospital-based provision, exhibited an intermediate resilience.

Overall, this study demonstrates how surgical resilience analysis can inform health policy and planning for future pandemics. These differentiated patterns suggest that targeted, procedure-specific strategies may be more effective than broad suspension policies, potentially reducing secondary health effects while maintaining essential infection control objectives.

## Figures and Tables

**Table 1:** Overall Surgery Volumes and Trends (2017–2021).

Year	Cataract Surgery (n)	Cataract YoY Growth (%)	Spine Surgery (n)	Spine YoY Growth (%)	Joint Replacement (n)	Joint YoY Growth (%)
2017	1,224,127	-	234,870	-	106,188	-
2018	1,336,494	9.2	241,359	2.8	110,554	4.1
2019	1,585,393	18.6	258,077	6.9	124,616	12.7
2020	1,661,027	4.8	271,101	5.0	121,048	−2.9
2021	2,330,589	40.3	288,901	6.6	127,400	5.2
5-Year Total Growth		90.4		23.0		20.0

**Table 2:** Interrupted Time Series Analysis of COVID-19 Pandemic Impact.

Surgery Type	Pre-Pandemic Growth (2018–2019, %)	Immediate Level Change (March 2020, %)	95% CI	Trend Change (Monthly, %)	Pandemic Year 1 (2020) Growth (%)	Recovery Year (2021) Growth (%)
Cataract	18.6	+2.1 (*p* = 0.003)	0.8–3.4	+1.8 (*p* < 0.001)	4.8	40.3
Spine	6.9	−1.2 (*p* = 0.141)	−2.8 – 0.4	+0.3 (*p* = 0.012)	5.0	6.6
Joint Replacement	12.7	−8.7 (*p* < 0.001)	−12.1 – −5.3	+0.7 (*p* = 0.002)	−2.9	5.2

**Table 3:** Regional Distribution of Surgeries with Pandemic Impact Analysis (2021).

Region	Cataract 2021 (n)	Cataract Δ20 (%)	Cataract Δ21 (%)	Spine 2021 (n)	Spine Δ20 (%)	Spine Δ21 (%)	Joint 2021 (n)	Joint Δ20 (%)	Joint Δ21 (%)
Gyeonggi-do	550,881	+10.1	+39.2	68,015	+11.5	+9.0	25,318	+4.4	+6.5
Seoul	401,929	+7.3	+32.4	42,868	+7.4	+6.5	19,847	−0.5	+2.9
Busan	195,354	+3.0	+48.0	18,103	+5.1	+3.4	9203	−2.9	+7.9
Gyeongnam	160,794	+5.1	+48.9	21,943	+8.3	+5.6	9250	−11.0	+2.3
Incheon	133,660	+15.6	+33.9	16,405	+9.3	−0.3	5974	−1.1	+4.4
Gyeongbuk	123,519	−6.1	+43.9	17,007	−5.1	+7.3	9150	−6.8	+2.1
Jeonnam	107,908	−3.7	+45.3	17,388	−2.9	+7.8	7856	−6.0	+5.1
Chungnam	102,545	−2.2	+42.3	15,302	−4.6	+9.0	6895	−9.6	+10.1
Capital Area *	1,086,470	+9.6	+36.0	127,288	+9.8	+6.8	51,139	+1.8	+4.9
National Total	2,330,589	+4.8	+40.3	288,901	+5.0	+6.6	127,400	−2.9	+5.2

* Capital Area includes Seoul, Gyeonggi-do, and Incheon. Δ values represent year-over-year percentage changes. Δ20 = 2019→2020; Δ21 = 2020→2021. Negative values are highlighted in red.

**Table 4:** Healthcare Facility Type Distribution (2019–2021).

Facility Type	Cataract 2019	Cataract 2020	Cataract 2021	Spine 2019	Spine 2020	Spine 2021	Joint 2019	Joint 2020	Joint 2021
Tertiary Hospital	107,239	93,117	126,077	45,372	46,453	53,592	16,834	16,914	19,639
General Hospital	112,962	110,629	124,165	72,694	80,655	83,539	51,688	51,715	53,192
Hospital	149,438	175,558	219,074	136,502	140,295	147,748	53,840	50,290	52,375
Clinic	1,215,237	1,281,282	1,860,841	3491	3667	3994	2254	2129	2195

Note: Values represent absolute surgical procedure counts by facility type across pre-pandemic (2019), pandemic (2020), and recovery (2021) periods.

**Table 5:** Correlation Analysis with COVID-19 Case Numbers.

Surgery Type	Correlation Coefficient (r)	*p*-Value	Interpretation	Clinical Implication
Cataract	0.23	0.045	Weak positive correlation	Resilient to pandemic disruption
Spine	−0.08	0.478	No significant correlation	Maintained stable volumes
Joint Replacement	−0.45	<0.001	Moderate negative correlation	Significantly affected by pandemic waves

## Data Availability

The datasets analyzed during the current study are not publicly available due to patient privacy and ethical restrictions but are available from the corresponding author upon reasonable request.

## Abbreviations

The following abbreviations are used in this manuscript:

CI	Confidence Interval
COVID-19	Coronavirus Disease 2019
IRB	Institutional Review Board
ITS	Interrupted Time-Series
KCD	Korean Classification of Diseases
NHIS	National Health Insurance Service
